# Rehabilitation of Severely Attrited Teeth with Hobo Twin Stage Technique: A Case Report

**DOI:** 10.31729/jnma.4745

**Published:** 2019-12-31

**Authors:** Anjana Maharjan, Sarita Joshi, Anand Verma, Ujjwal Rimal

**Affiliations:** 1 Patan Academy of Health Sciences, Patan, Nepal; 2 National Academy of Health Sciences, Kathmandu, Nepal; 3 Kathmandu University School of Medical Sciences, Dhulikhel, Kavre, Nepal

**Keywords:** *attrited*, *hobo*, *occlusion*, *rehabilitation*

## Abstract

Restoration of excessively worn dentition is a challenging treatment procedures. It requires efficient diagnosis and treatment plan. Hobo's techniques and Pankey Mann Schuyler's philosophy are widely used and documented for full mouth rehabilitation. We have reported the case of a 56-year-old male patient who presented with the severely worn dentition and had difficulty in chewing. To rehabilitate this case Hobo's twin stage technique had been adopted as it is based on scientific data and mathematical analysis for both disocclusion and anterior guidance thus reducing chair side time.

## INTRODUCTION

Gradual wear of occlusal surfaces of teeth is a normal process but excessive wear of teeth results in compromised esthetics and impaired function. Excessive wear of the occlusal surface of teeth leads to the decreased vertical dimension of occlusion (VDO).^1^

Full mouth rehabilitation is a challenging treatment modality dealing with reconstruction and restoration of the worn dentition and maintenance of the stomatognathic system.^2^ The indications for occlusal rehabilitation include the following condition-the restoration of multiple teeth which are broken, worn, missing or decayed, faulty fixed partial dentures work, discolored dentition, developmental defects and worn out dentition.^3^ Hobo's techniques and Pankey Mann Schuyler (PMS) philosophy are widely used and documented for full mouth rehabilitations.^4^

This case report presents the procedures of rehabilitation of a patient with severely worn dentitions with decreased VDO. The rehabilitation of this patient includes all-ceramic crowns and implant restorations with increasing the vertical dimensions of occulusion using the Hobo's twin stage technique. The patient's consent had been taken for this publication.

## CASE REPORT

A 56-years-old male patient reported to the department of prosthodontics NAMS, Bir Hospital with severely worn teeth. He gave the history of chewing hard food like betel nuts, hard cheese and bone which could be the cause for the severely worn dentition. The patient was expalined about the consequences of his habit and advised to stop the habits.

Clinical and radiographic examinations revealed severe tooth surface loss on the mandibular and maxillary anterior and posterior teeth and uneven occlusal surfaces.

While evaluating esthetics: the face was symmetrical with a straight profile and nasolabial angle less than 90°, facial midline coincides with dental midline, lips were symmetrical and competent with 50% mandibular teeth visible short lip length i.e 20mm.

**Figure 1:**
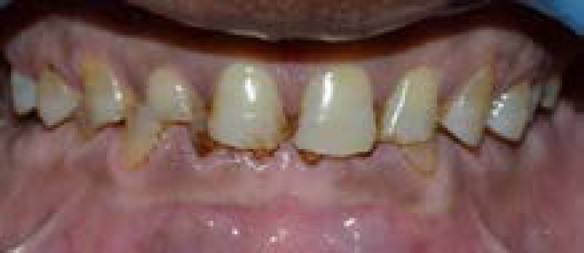
Preoperative Intraoral view at occlusion and upper and lower occlusal view.

The vertical dimension was determined by Niswonger's Thomoson's technique: vertical dimension at occlusion (VDO) was 52mm and vertical dimension at rest (VDR) was 60mm suggesting free way space of 8mm approximately (Figure 1).

**Figure 2:**
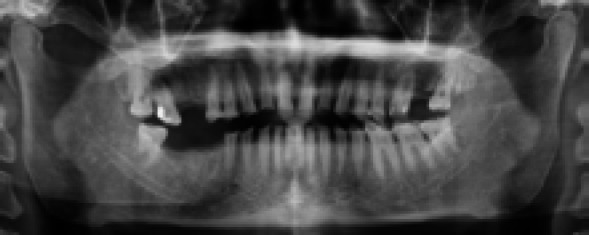
Preoperative Orthopantomogram.

After obtaining written informed consent, treatment was carried out with diagnostic casts from primary impressions made with alginate (Troicalgin, Zhermark, Rovigo, Italy). Orientation jaw relation was recorded using a face bow (Hanau spring bow) and centric relation was recorded by Dawson's technique. These records were then transferred to a semi-adjustable articulator (Hanau Wide Vue).

The existing VDO was increased by 4mm using the incisal guidance pin of the articulator to a new VDO of 56mm. An occlusal splint was fabricated at the increased vertical dimension, The splint maintained uniform tooth contact in centric relation (CR) and disocclusion of posterior teeth in an eccentric movement.

After endo consultation the patient went through endodontic treatment of 12,13,15,17,26,31,35,41,43 and 44. The adaptation of this new increased VDO was evaluated for a period of 1 month using an occlusal splint, during this period, temporomandibular joint discomfort and muscle tenderness were not found. Diagnostic wax-up was done maintaining the increased VDO on the mounted diagnostic cast. Putty index was then made. Initial tooth preparation on 11,12,13,14,15, 17,21,22,23,23,24,25,26,31,32,33,34,35,41,42,43,44,45,46 and 47 was done for all-ceramic restoration with equigingival heavy chamfer finish line. Impression was made and the cast was poured. Acrylic resin temporary crown was fabricated using an indirect technique. Esthetics and phonetics were evaluated with the provisional fixed restorations prior to the cementation with provisional luting cement (Freegenol Temporary Pack; GC Corp., Tokyo, Japan).

Adaptation of these provisional restorations was evaluated for three weeks. After which final tooth preparation and impressions were made with polyvinyl siloxane (Reprosil;Densply,USA). The maxillary cast was mounted with the face bow record and centric relation was recorded with the help of Lucia jig with the anterior provisional in place.

The articulator was programmed to condition 1 of Hobo's twin stage procedure (Table 1).

**Table 1:** Values of condition 1 and 2 according to the Hobo twin- stage technique (values in degrees).^5^

	Horizontal condylar guidance	Lateral condylar guidance	Anterior guidance	Lateral condylar guidance
Condition 1	25	15	25	10
Condition 2	40	15	45	20

Posterior wax-up was done in relation to the values of condition 1 (Hobo twin stage technique) and anterior wax-up was done in relation to condition 2. After verification of posterior disocclusion in protrusive and laterotrusive movements the wax patterns were sent for the fabrication of all ceramic restoration.

These all-ceramic restorations were cemented with resin cement (Calibra; Dentsply) after etching of all-ceramic crown (lithium disilicate) with Hydroflouric acid(Ivoclar; Etching gel) 5% for 20 seconds and application of silane coupling agent (Ivoclar; Monobond N) (Figure 4a and 4b). Two Dio implants of 4.5×10mm were placed in relation to 46 and 47 region.

After a few month's time, the crown in relation to 45 got dislodged along with a fractured coronal third of root. Hence an immediate implant (3.5×8.0 mm) with immediate provisionalization was given.

**Figure 3a and 3b:**
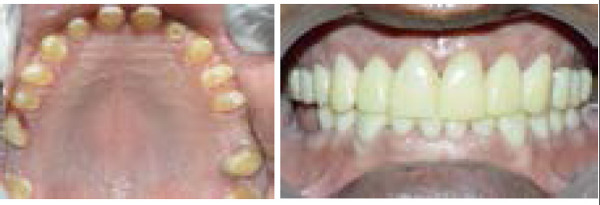
Teeth Preparation done to receive individual crown and bridge followed by provisional restoration.

**Figure 4a and 4b:**
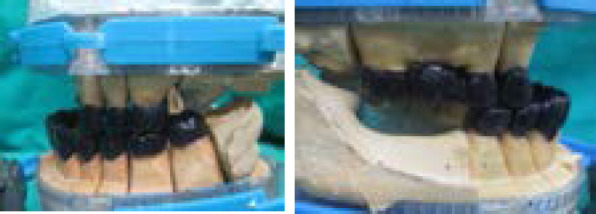
The anterior segment of the maxillary cast is removed for waxing up the posterior teeth.

**Figure 5a and 5b:**
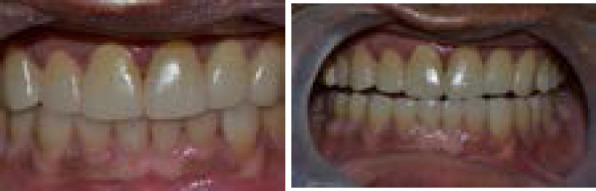
Final prosthesis inserted and centric and eccentric contact verified.

**Figure 5c:**
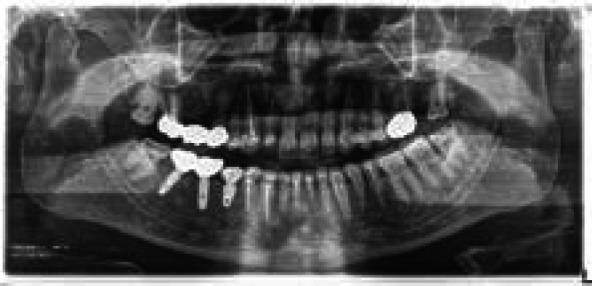
Postoperative Orthopantomogram.

## DISCUSSION

The vertical dimension of occlusion (VDO) is maintained even after rapid wear because of compensatory alveolar process elongation by progressive remodeling of the alveolar bone.^6^ However, occlusal wear may occur more rapidly than continuous eruption depending on the etiology of the wear. Excessive teeth wear causes loss of Vertical dimension (VD) which leads to decreased esthetic, discomfort while chewing ultimately decreasing the quality of life.^7^

It is important to establish the cause of wear before the intervention to help improve the effectiveness of any preventive and restorative care.^8^ VD lost can be verified by a combination of various techniques such as phonetics, esthetics, interocclusal distance.^9^ In our case, approximately 4 mm of loss of VDO was established and the amount of bite raise to be achieved was evaluated using the “closest S - speaking space” and the amount of “Freeway space” .

According to Turner's category -1 excessive wear with loss of VD, removable splint for 6-8 weeks with increased VD should be given to the patient followed by fixed provisional restoration for 2-3 weeks before planning permanent restoration.^10^

As suggested by Turner, we had adapted similar techniques to increase VD and stabilization of occlusion using heat-cured provisional restoration for a 3 weeks.

There are various techniques during full mouth rehabilitation, whether to work on different segments of the arch individually or full arch preparation. In our case, we had utilized the quadrant wise preparation and temporization in order to maintain VD, quadrant anesthesia and shorter appointments. This technique also has disadvantages like restrictions of achieving ideal occlusion while altering VD, occlusal plane.

Thus, full mouth rehabilitation is a challenging procedure involving complicated clinical and laboratory steps. The twin stage technique formulated by Hobo and Takayama reproduces disocclusion and anterior guidance more precisely and scientifically.^11^ In this case we have utilized the same procedure to achieve targeted esthetics and function.

## Consent:

**JNMA Case Report Consent Form** was signed by the patient and the original is attached with the patient's chart.

## Conflict of Interest

**None.**
